# Does the introduction of streamlight decrease refractive surgery operating time?

**DOI:** 10.1007/s10792-024-03093-x

**Published:** 2024-04-18

**Authors:** Piotr Kanclerz, Katarzyna Przewłócka, Michael Mimouni

**Affiliations:** 1 ArtLife Ophthalmological Center, ul. Obrońców Wybrzeża 23, 80-398 Gdańsk, Poland; 2 Department of Ophthalmology, Hygeia Clinic, Gdańsk, Poland; 3 Helsinki Retina Research Group, University of Helsinki, Helsinki, Finland; 4 Department of Ophthalmology, Rambam Health Care Campus Affiliated with the Bruce and Ruth Rappaport Faculty of Medicine, Technion-Israel Institute of Technology, Haifa, Israel

**Keywords:** Alcohol-assisted photorefractive keratectomy, Excimer laser, Laser vision correction, Streamlight, Transepithelial photorefractive keratectomy

## Abstract

**Purpose:**

The aim of the study was to analyze the time-savings associated with introduction of Streamlight™ (Alcon Laboratories, Fort Worth, TX, USA) transepithelial photorefractive keratectomy (PRK) in surface corneal ablations.

**Methods:**

All refractive surgeries were performed using the Alcon WaveLight® EX500 at the ArtLife Clinic, Gdańsk, Poland. The study included patients treated for refractive errors with transepithelial PRK between April 2019 and October 2021, who were matched with patients treated with alcohol-assisted PRK during the same period. Only results for the left eye were analyzed.

**Results:**

One-hundred-five patients underwent transepithelial PRK (age 33.42 ± 8.67 years) and were matched with 105 patients that underwent alcohol-assisted PRK (age 33.05 ± 10.16 years; *p* = 0.11). The mean preoperative spherical equivalent refraction was − 2.04 ± 2.28 D, and − 1.9 ± 1.71 D for the transepithelial and alcohol-assisted PRK group, respectively (*p* = 0.20). The total surgery time was non-significantly shorter in transepithelial PRK (349.46 ± 47.83 s) than in alcohol-assisted PRK (354.93 ± 137.63 s; *p* = 0.7); however, the variance of surgical time was significantly lower in transepithelial PRK (*p* < 0.001). The laser treatment duration was greater in transepithelial PRK (41.78 ± 17.2 s) than in alcohol-assisted PRK (8.48 ± 6.12 s; *p* < 0.001), and so was the number of breaks during the laser treatment (0.95 ± 0.63 vs. 0.53 ± 0.88, respectively; *p* < 0.001).

**Conclusion:**

The introduction of transepithelial PRK did not bring significant time-associated savings into the refractive surgery suite.

## Introduction

Corneal refractive surgery was introduced more than thirty years ago [1]. After several years of development and innovations in excimer lasers, today we can use state-of-the-art devices with high-speed procedures, fast eye-trackers, pupillary monitoring systems, and advanced customization profiles [2], that allow for minimally invasive refractive surgery procedures. These devices are capable of providing superior treatments with excellent postoperative visual acuity and can decrease higher-order aberrations. Currently, the American Academy of Ophthalmology states that excimer laser refractive surgery, whether it is a surface or laser in situ keratomileusis procedure, is a safe and effective method of correcting a wide range of refractive errors with some limitations for high ametropia and astigmatism [3].

One of the recently developed modalities of refractive surgery is the single-step transepithelial photorefractive keratectomy (PRK). The concept of transepithelial PRK is not new, as it was introduced in the 1990s by Alio et al. [4] In this procedure, the epithelium is removed by laser (phototherapeutic keratectomy), followed by PRK; this prevents the necessity for epithelial removal chemically (using alcohol) or mechanically (using a blade or brush). Historically, the adoption of this two-step approach was limited by no uniform way to perform the procedure, more difficult planning and hyperopic shift [5]. The single-step approach introduced with the new laser software minimizes the risk of dehydration and reduces the energy levels used to remove the epithelium - in order to prevent the hyperopic shift [6]. The diameter, and the volume of the removed epithelium are precisely software controlled. Transepithelial PRK is available in the Amaris 1050RS (SCHWIND eye-tech-solutions GmbH, Kleinostheim, Germany), the Technolas Teneo 317 (Technolas Perfect Vision GmbH/Bausch + Lomb, Munich, Germany), and the Wavelight EX500 Platform (Alcon Laboratories, Fort Worth, TX, USA). Streamlight™ is the trademark for transepithelial PRK within the Wavelight EX 500; in this single-program, the epithelium and then stroma are ablated to achieve a planned refractive outcome.

Employing transepithelial PRK not only simplifies the procedure by restricting the number of surgical steps, but could potentially allow the surgeon to perform a continuous surgical session in a shorter time [7]. The aim of the study was to analyze the time-savings associated with the introduction of Streamlight™ transepithelial PRK compared with alcohol-assisted PRK.

## Materials and methods

This study was conducted based on data from the ArtLife Clinic, Gdańsk, Poland; this practice focuses mainly on corneal refractive surgery, namely surface ablations, and microkeratome-assisted LASIK procedures. All refractive surgeries are performed using the WaveLight® EX500 excimer laser. Starting in early 2019, Streamlight™ was introduced as a software update into the device. The decision on whether the epithelium should be debrided manually or with the laser platform was made by the surgeon together with the patient having considered the benefits and drawbacks of each method. For the purpose of this study, medical records of patients undergoing Streamlight™ between April 2019 and October 2021 were included. The results were compared with age- and refractive error-matched patients that underwent bilateral alcohol-assisted PRK in the same period and were by the same surgeon (P.K.).

Prior to the surgery, all patients had a complete ophthalmologic examination consisting of subjective refraction, noncontact tonometry, non-cycloplegic and cycloplegic refraction, slit lamp evaluation, ophthalmoscopy, and corneal tomography with the Alcon WaveLight® Oculyzer. Visual acuity is presented as Snellen decimal fractions. Refractive errors were classified as recommended by the International Myopia Institute [8] and the cylinder power was presented as negative values. Astigmatism was defined as cylinder power equal or greater than 0.75 D [9]. Both myopic and hyperopic cases were included, as Streamlight™ allows correction of hyperopia up to + 3.0 Diopters.

Surgery time was calculated by retrospective analysis of the reports from the WaveLight EX500 laser. Since it is known that using a combined measurement from both eyes is likely to underestimate the true variance of a sample [10, 11], only results for the left eye were used. All surgeries were conducted bilaterally, and the right eye was treated first. The time of surgery was calculated by subtracting the time of the surgery conclusion for the left eye (e.g., 11:36:21) from the time of the conclusion of the surgery of the right eye (e.g., 11:30:05). Duration of laser treatment, as well as the number of breaks during treatment, was recorded. Only the total surgery time per one eye but not the total operating room time was analyzed. This approach aimed to minimize the impact of potential confounding factors, such as comorbidities or communication challenges (e.g., when a patient did not speak Polish language) which would rather affect the total operating room, but not the subtraction time.

All procedures were performed under topical anesthesia, with proxymetacaine hydrochloride 0.5% (Alcaine) applied three times before the surgery to each eye. Streamlight™ uses the excimer laser to remove most of the epithelium, and the cornea was dried before epithelium removal. The depth of laser epithelium removal was set to 55 µm, and if the surgeon suspected that some epithelium might be left, the rest was manually removed before PRK was performed. In all alcohol-assisted PRK cases, 20% alcohol was poured into a well, and positioned over the center of the cornea for 30 s. In single patients in whom it was not possible to place the well due to eye movements or lack of cooperation, 20% alcohol was placed on a circular sponge for 30 s. Subsequently, the epithelium was removed manually [12]. All ablations were conducted using the wavefront-optimized profile with an optical zone of 6.5 mm. All patients postoperatively received moxifloxacin 3 times a day for 7 days, dexamethasone four times a day for a minimum of 4 weeks (based on intraocular pressure and visual function), and lubrication with hyaluronic acid 6–8 times a day for the first week (and in the following weeks at least four times a day) [3, 13]. A bandage contact lens was placed for seven days following surgery [13]. The study adhered to the tenets of the Declaration of Helsinki for the use of human participants in biomedical research and was approved by the local research ethics board. As this was a retrospective database study, it was exempted from the requirement to receive approval from the Local Bioethical Committee (*Komisja Bioetyczna Przy Izbie Lekarskiej w Gdańsku)*.

Statistical analysis was performed using MedCalc Statistical Software version 14.8.1 (MedCalc Software bvba, Ostend, Belgium) and IBM SPSS Statistics v. 28 (IBM Corporation, Armonk, NY, USA). To assess the normality distribution of data we used the Shapiro-Wilko test. For analyzing differences in normally distributed data, a two-tailed t-test was applied and the data were presented as mean ± standard deviation (SD). The F-test was used to assess the equality of variances. In cases of an unequal variance the Welch-test was performed to compare data, as the most reliable and accurate method to compare central tendency for two unrelated samples. Differences among categorical data were analyzed using the Chi-Square test. Multivariate logistic regression analysis was used to analyze the association between total surgical time and gender, age and spherical equivalent refraction (SER). Sample size calculation was performed using the PS program (version 3.0) for power and sample size calculation [14]. A sample size of 105 eyes per group was estimated to detect a difference in surgical time of 5 s, based on an SD of difference between the surgical time of 10 s, a power of 95% at a significant level of 5%. Results with *p* levels under 0.05 were considered as statistically significant.

## Results

One-hundred-five patients underwent transepithelial PRK with an average age of 33.42 ± 8.67 years. They were matched with 105 patients who underwent alcohol-assisted PRK, with an average age of 33.05 ± 10.16 years (*p* = 0.11). The most common indication for surgery in both groups was myopia and myopic astigmatism (Table 1). The mean preoperative spherical equivalent refraction was − 2.04 ± 2.28 D for the transepithelial PRK group and − 1.9 ± 1.71 D for the alcohol-assisted PRK group (*p* = 0.20). The mean preoperative uncorrected distance visual acuity was non-significantly better in the transepithelial PRK than in the alcohol-assisted PRK group (0.30 ± 0.15 vs. 0.02 to 0.90; *p* = 0.06), but there was no difference in corrected distance visual acuity (0.90 ± 0.09 vs. 0.93 ± 0.09; *p* = 0.58).

**Table 1:** Baseline characteristics of patients undergoing transepithelial and alcohol-assited photorefractive keratectomy

	Transepithelial PRK (*n* = 105)	Alcohol-assisted PRK (*n* = 105)	*p*-value
Age [Years]	33.42 ± 8.67	33.05 ± 10.16	0.11
	[19 to 60]	[19 to 65]	
Gender			0.24
Female	51.4% ( = 54)	46% (*n* = 48)	
Male	48.6% (*n* = 51)	54% (*n* = 57)	
Indication for surgery:			
Preoperative Uncorrected Distance Visual Acuity	0.3 ± 0.15	0.25 ± 0.31	0.06
	[0.10 to 0.80]	[0.02 to 0.90]	
Preoperative Spherical Equivalent Refraction [D]	− 2.04 ± 2.28	− 1.9 ± 1.71	0.2
	[− 7.50 to 4.50]	[− 5.75 to 5.25]	
Myopia	43.81% (*n* = 46)	42.86% (*n* = 45)	
Myopic astigmatism	36.19% (*n* = 38)	39.05% (*n* = 41)	0.95
Mixed astigmatism	8.57% (*n* = 9)	9.52% (*n* = 10)	
Hyperopia	5.71% (*n* = 6)	4.76% (*n* = 5)	
Hyperopic astigmatism	5.71% (*n* = 6)	3.81% (*n* = 4)	
Preoperative Refractive Cylinder [D]	− 1.14 ± 1.23	− 0.87 ± 0.90	0.9
	[− 15.00 to 3.00]	[− 5.75 to 5.25]	
Preoperative K1 [D]	42.81 ± 1.42	42.75 ± 1.6	0.27
	[39.30 to 46.40]	[38.30 to 47.40]	
Preoperative K2 [D]	44.38 ± 1.47	43.96 ± 1.66	0.08
	[40.40 to 47.90]	[39.90 to 47.80]	
Preoperative pachymetry [μm]	545.66 ± 32.03	546.17 ± 33.49	< 0.001
	[466.00 to 628.00]	[469.00 to 516.00]	
Preoperative Best Corrected Visual Acuity [SnDec]	0.9 ± 0.09	0.93 ± 0.09	0.58
	[0.80 to 0.90]	[0.70 to 1.00]	

K—keratometry, PRK—photorefractive keratectomy; values presented as mean ± standard deviation. Range of data presented in brackets

**Fig. 1:**
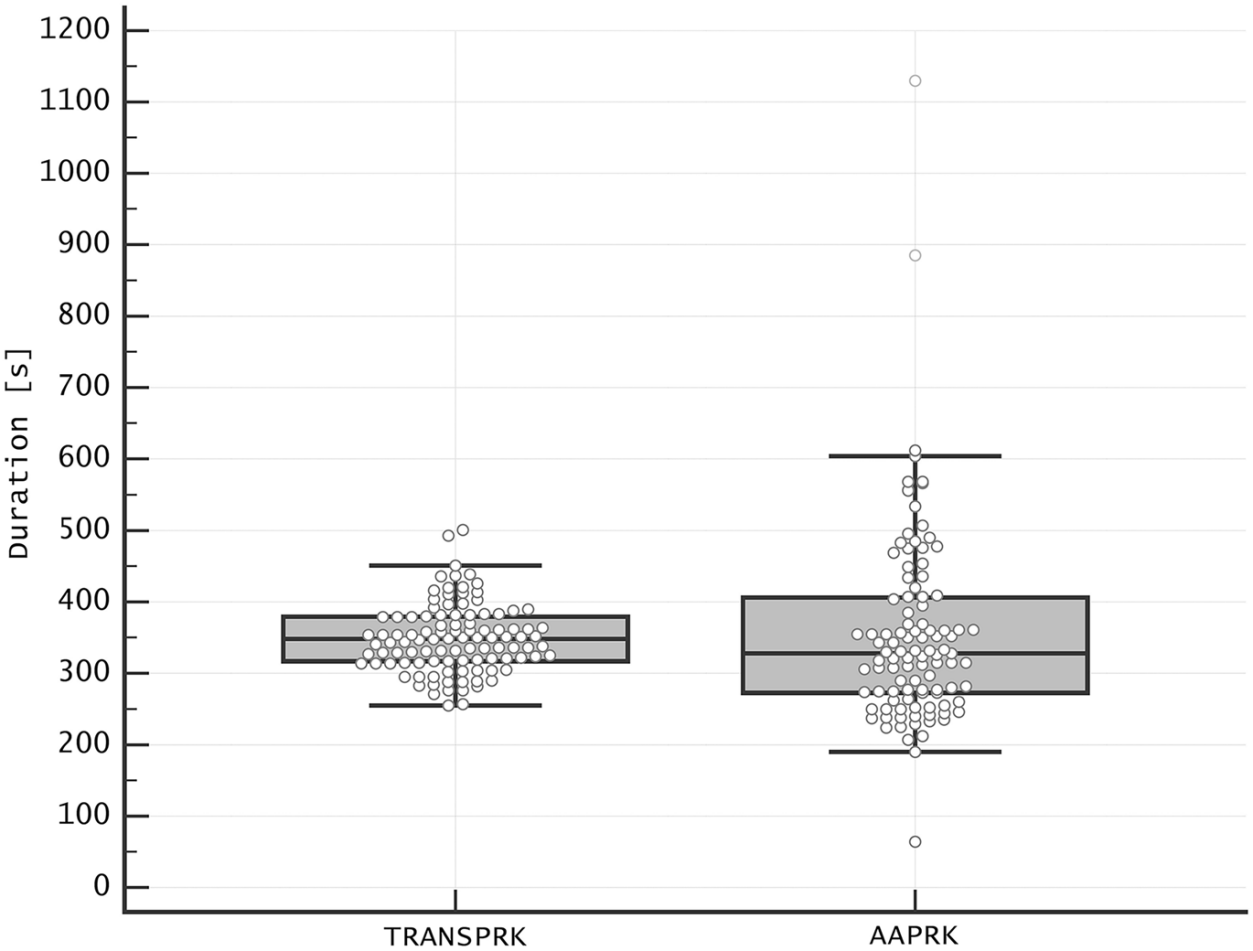
Total surgery duration time in transepithelial photorefractive keratectomy (TRANSPRK) and alcohol-assisted photorefractive keratectomy (AAPRK)

The total surgery time of the left eye was non-significantly shorter in transepithelial PRK than in alcohol-assisted PRK (349.46 ± 47.83 s vs. 354.93 ± 137.63 s; *p* = 0.7; Fig. 1). The variance of the surgical time was lower in transepithelial PRK than in alcohol-assisted PRK (2,287.71 s^2^ vs. 18,942.02 s^2^; *p* < 0.001). The laser treatment duration was greater in transepithelial PRK than in alcohol-assisted PRK (41.78 ± 17.2 vs. 8.48 ± 6.12 s; *p* < 0.001), and so was the number of breaks during the laser treatment (0.95 ± 0.63 vs. 0.53 ± 0.88; *p* < 0.001; Table 2). The stromal ablation depth for transepithelial PRK was 35.52 ± 39.70 μm, while 33.08 ± 29.78 μm for alcohol-assisted PRK (*p* = 0.61).

**Table 2:** Total duration of surgery and laser treatment in alcohol-assisted and transepithelial photorefractive keratectomy (PRK)

	Transepithelial PRK (*n* = 105)	Alcohol-assisted PRK (*n* = 105)	*p*-value
Laser treatment duration [s]	41.78 ± 17.2	8.48 ± 6.12	< 0.001
	[23.00–165.00]	[2.00–43.00]	
Number of breaks during laser treatment	0.95 ± 0.63	0.53 ± 0.88	< 0.001
	[0–5]	[0–4]	
Total surgery time [s]	349.46 ± 47.83	354.93 ± 137.63	0.7
	[64.00–1130.00]	[255.00–501.00]	
Stromal ablation depth [μm]	35.52 ± 39.70	33.08 ± 29.78	0.61
	[8.0–130.0]	[10–116]	

Multivariate linear regression demonstrated that the total surgery time was associated with the surgical method (*p* < 0.001), marginally negatively correlated with age (β =  − 0.058; *p* = 0.05), but not with gender (*p* = 0.12) and SER (*p* = 0.17).

## Discussion

This study has shown that the duration of surgery is not statistically different in transepithelial PRK and alcohol-assisted PRK. Alcohol delamination of the corneal epithelium before PRK routinely leaves in a very smooth cleavage at the area of the hemidesmosomal attachments, including the superficial lamina lucida [15]. Thus, the smooth surface left after alcohol delamination can be easier and faster and probably more comfortable for the surgeon than mechanical debridement. The variance of surgical time was significantly lower in transepithelial than in alcohol-assisted PRK; this could translate into a more predictable surgery duration and easier operating room planning. A study by Zarei-Ghanavati et al. has shown a difference in the operation time in transepithelial vs. alcohol-assisted PRK using the Amaris 750S platform (34.0 ± 7.6 s vs. 44.5 ± 12.1 s, respectively; *p* = 0.000) [16]. Another study from Saudi Arabia has found a greater difference in surgical time between transepithelial and alcohol-assisted PRK using the Schwind Amaris 750S (162.2 ± 14.8 s vs. 243.2 ± 98.7 s, respectively; *p* < 0.001) [17]. The difference between the two methods is more prominent than in our study, and this could be related to surgical technique and with surgeon’s-related factors. Moreover, two surgeons conducted the operations in the aforementioned study; their experience was not clarified, and their operating time was not compared. Another study also found relevantly shorter surgical time using the Amaris platform in transepithelial PRK vs. PRK with manual debridement of the epithelium with a blunt spatula (58.0 ± 6.4 s vs. 98.6 ± 9.8 s, respectively) [18]. A significant limitation of the previously published studies is that they do include a precise definition of operation time, or present information on the methodology. A strength of our study is that the methodology and study design is very precise and clear.

The main limitation of this study was that it evaluated procedures performed by one surgeon. One could expect that for other surgeons, having other experience or surgical techniques, the results may vary. Moreover, the time of surgery for other surface ablation techniques e.g., epi-Bowman keratectomy presumably could be different due to other surgical steps during treatment. Interestingly, when evaluating median times of surgery, the surgical time of alcohol-assisted PRK was slightly shorter than that of transepithelial PRK. Although this has not been documented and analyzed, this could be associated with long surgery times in some non-cooperative patients, in whom it was not possible to steadily place the alcohol well, and the alcohol-soaked sponge was used. Moreover, this did not allow such an easy removal of the epithelium and could have resulted in significantly longer epithelial scraping; in these cases, the surgery could have lasted longer. On the other hand, the number of breaks during laser treatment was greater in transepithelial PRK than in alcohol-assisted PRK (0.95 ± 0.63 vs. 0.53 ± 0.88). This was probably associated with more common loss of fixation during longer treatments, but not necessarily influenced the surgical time.

We might conclude that the introduction of Streamlight™ did not bring significant time-associated savings into the refractive surgery suite.

### What was known

- Excimer laser refractive surgery is a safe and effective method of correcting a wide range of refractive errors.
- Transepithelial photorefractive keratectomy (PRK) simplified the procedure by restricting the number of surgical steps.

### What this paper adds

- The introduction of Streamlight™ does not bring significant time-associated savings into the refractive surgery suite.
- The variance of the surgical time was significantly greater in alcohol-assisted PRK than in transepithelial PRK.
